# Effects of Tai Chi Chuan on cognitive function in adults 60 years or older with mild cognitive impairment: a systematic review and meta-analysis

**DOI:** 10.3389/fphys.2025.1556622

**Published:** 2025-04-01

**Authors:** Wen-Ting Wang, Hong Wang

**Affiliations:** ^1^ School of Graduate, Wuhan Sports University, Wuhan, China; ^2^ Department of Sports Tourism and Foreign Languages, Henan Sport University, Zhengzhou, China; ^3^ School of Wushu, Wuhan Sports University, Wuhan, China

**Keywords:** Tai Chi Chuan, older adults, mild cognitive impairment, global cognition, memory, executive function

## Abstract

**Background:**

Mild cognitive impairment (MCI) is an intermediate stage between normal aging and dementia. Emerging evidence has demonstrated that mind-body interventions may enhance cognitive function. To elucidate whether stand-alone Tai Chi Chuan (TCC) intervention confers domain-specific benefits on executive function, memory, and global cognition, further investigations should be conducted.

**Purpose:**

This systematic review and meta-analysis of randomized controlled trials (RCTs) examined TCC’s effects on global cognition, memory, and executive function, and its duration-response relationship in adults 60 years or older with MCI.

**Methods:**

Seven electronic databases were searched for relevant literature, with English as the sole inclusion criterion for language. The methodological quality and risk of bias for all included RCTs were assessed using the Cochrane Risk of Bias (2.0) tool. The pooled effect sizes were evaluated using standardized mean differences (SMD) and 95% confidence intervals (CI). A p-value <0.05 was considered statistically significant.

**Results:**

Nine of the 1,442 publications met the inclusion criteria, comprising RCTs involving 1,066 participants (68.95% female) with a mean age of 74.1 (±7.4) years. Long-term TCC demonstrated significant effects on global cognition (p < 0.001; SMD = 0.488; 95% CI: 0.222–0.754), whereas short-term TCC did not (p = 0.172; SMD = 0.682; 95% CI: −0.397–1.660). Overall, TCC showed significant global cognitive benefits (p = 0.003; SMD = 0.526; 95% CI: 0.184–0.869). Long-term memory showed no improvement (p = 0.214; SMD = 0.162; 95% CI: −0.094–0.417), while short-term memory improved significantly (p = 0.021; SMD = 1.010; 95% CI: 0.155–1.865). The overall effect of TCC on memory was significant (p = 0.005; SMD = 0.580; 95% CI: 0.178–0.982). Both short-term and long-term improvements in executive function were significant (p = 0.006; SMD = −0.791; 95% CI: −1.353 to −0.230).

**Conclusion:**

The study confirmed TCC’s duration-dependent effects on global cognition in older adults (≥60 years) with MCI. Memory exhibited nonlinear temporal dynamics, characterized by short-term acceleration and long-term plateau, while executive function demonstrated temporal invariance with comparable efficacy across intervention durations.

**Systematic Review Registration:**

https://www.crd.york.ac.uk/PROSPERO/home, identifier CRD42024587754.

## Introduction

In 2020, the World Health Organization and the United Nations designated the period 2021–2030 as the Decade of Healthy Ageing (Cacchione, 2022). With global population aging, addressing cognitive decline in older adults has become a pressing public health priority (Dunne et al., 2021). The progression from cognitive decline to dementia encompasses three clinical stages: diabetes-related cognitive impairment, MCI, and dementia. Updated guidelines from the American Academy of Neurology (2018) report age-stratified MCI prevalence: 6.7% (60–64 years), 8.4% (65–69), 10.1% (70–74), 14.8% (75–79), and 25.2% (80–84) (Petersen et al., 2018). MCI is characterized by progressive memory loss or cognitive dysfunction that neither significantly impairs daily living activities nor meets diagnostic criteria for Alzheimer’s disease (AD). As a transitional precursor to AD, MCI represents an unstable state with elevated dementia conversion risk. Longitudinal studies indicate a 14.9% cumulative incidence of dementia among individuals with MCI aged ≥ 65 years over 2-year follow-up periods. Current evidence does not support pharmacological interventions for MCI. However, non-pharmacological approaches show promise: 6-month exercise regimens may improve cognitive metrics, and cognitive training demonstrates potential therapeutic effects (Petersen et al., 2018).

The COVID-19 pandemic continues to have a substantial effect on global health (Kercher et al., 2022). Currently, inadequate physical exercise is linked to the most prevalent lifestyle-related chronic diseases (Batrakoulis, 2022a; Blair, 2009). The Exercise is Medicine initiative posits that physical activities can serve as evidence-based exercise prescriptions for certain patients, highly effective in improving their prognoses (Newsome et al., 2025). Despite being a physical exercise, TCC can foster both mental and physical contact. Mind-body fitness effectively guide the body to enter a state of calmness and relaxation by achieving a dynamic balance between body posture and respiratory regulation (Wells et al., 2012; Batrakoulis, 2022b). Within the United States, mind-body fitness such as TCC and Qigong are demonstrating a discernible upward trend (Clarke et al., 2018; Batrakoulis, 2022c). Currently, more than 300 million TCC enthusiasts are distributed across over 150 countries worldwide (Chen Zhu, 2024). Fourteen countries have conducted studies on the beneficial effects of TCC on a diverse range of diseases and overall physical wellbeing (Hong et al., 2024).

Current studies have confirmed that TCC exerts specific neuromodulatory effects on patients with MCI by regulating brain regions associated with cognitive control (e.g., enhanced prefrontal cortex function) (Tianyu and Li, 2024). Clinical research demonstrates that TCC combined with transcranial direct current stimulation (tDCS) significantly enhances global cognition, memory, executive function and attention in MCI patients (Xu et al., 2023). Meta-analyses further validate the efficacy of TCC in delaying cognitive deterioration across populations with varying cognitive statuses (Takemura et al., 2024; Wayne et al., 2014). Cognitive functioning comprises multiple interdependent domains, including (but not limited to) attention, memory, global cognition, decision-making processes, and executive function. The inherent characteristics of TCC—requiring sustained intentionality and conscious regulation of complex postural sequences—necessitate continuous engagement of three core cognitive systems: attention, memory, and executive control for postural balance (Li, 2014). Although research on mind-body interventions for MCI has surged over the past decade (Zhang et al., 2024), significant evidence gaps persist: (1) the absence of systematic reviews quantifying TCC’s differential effects across specific cognitive domains; and (2) ongoing controversies regarding the cognitive-enhancing efficacy of short-term, single-modality exercise interventions (Brasure et al., 2018). These limitations underscore the necessity to deepen investigations into TCC’s domain-specific cognitive effects, thereby providing high-level evidence to inform the development of stepped-care intervention strategies (e.g., dose-time optimization) for MCI.

To elucidate the duration-response relationship, this study implemented two key methodological decisions: (a) the use of TCC as the experimental intervention; and (b) stratification of practice duration into short-term (≤6 months) and long-term (>6 months) cohorts. This design enables simultaneous evaluation of: (a) TCC’s differential impacts on specific cognitive domains; and (b) the effect of intervention duration on cognitive improvement.

## Methods

### Study protocol and registration

This systematic review followed the recommendations of the Preferred Reporting Items for Systematic Reviews and MetaAnalyses (PRISMA) (Page et al., 2021) and the Cochrane Handbook for Systematic Reviews of Interventions. The protocol was registered in the International Prospective Register of Systematic Reviews (CRD: 42024587754; dates: first submission on 6 September 2024; PROSPERO registration 17 September 2024). The literature search was conducted from database inception through 1 September 2024.

### Search strategy

The literature search encompassed seven electronic databases from their inception to 1 September 2024, including Web of Science, PubMed, Cochrane Library, Embase, CINAHL Complete (EBSCO), MEDLINE (EBSCO), and China National Knowledge Infrastructure (CNKI). The search terms “Tai Chi Chuan” and “Mild Cognitive Impairment” were used to retrieve studies on cognitive function. These terms were employed in combination with their Medical Subject Headings (MeSH) terms, keywords, and synonyms. For the Chinese database (CNKI), the simplified Chinese characters “太极” (Tai Chi), “太极拳” (Tai Chi Chuan), and “轻度认知功能障碍” (MCI) or “认知障碍” (Cognitive Impairment) were used. Additional references were identified through manual searches of relevant literature.

### Eligibility criteria

Only available RCTs were included in the analysis. The inclusion criteria included: (1) the participants were adults 60 years or older, (2) only MCI without dementia or other disease that affected cognitive function, (3) Clinical Dementia Rating global score of 0.5 or lower, (4) clinically significant cognitive impairment (a score of ≥24 on the Mini-Mental State Evaluation; a score of ≥26 on the Montreal Cognitive Assessment Scale), (5) the experimental group only received TCC intervention without limiting time, frequency, form and place, (6) the control group was not restricted, (7) the results were more than primary outcomes.

### Literature search

Seven databases yielded 1,441 articles, with one additional record identified through manual searching.

**FIGURE 1:**
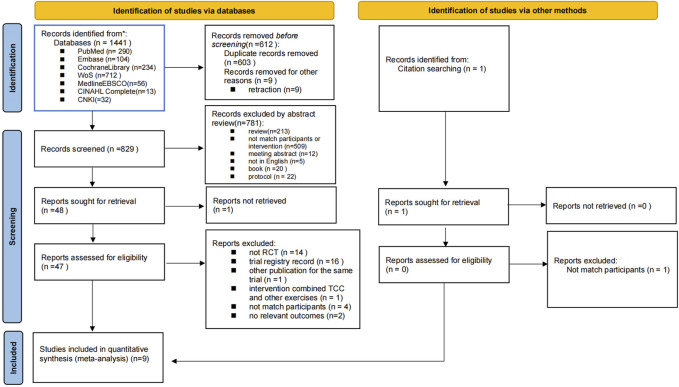
Flow diagram of the study selection process.

Using EndNote X9, 612 records were excluded during the pre-screening phase due to retracted publications or duplicates. Subsequently, 820 studies were excluded for not meeting the inclusion criteria, including one study that involved subjects with cognitive frailty instead of MCI. Figure 1 illustrates the study selection process.

### Participant characteristics and study setting

A total of 1,066 participants were included across the nine eligible studies. Geographical distribution of the studies included six conducted in China, one in the United States (US), and two in Thailand. Regarding study settings, eight studies focused on community-dwelling populations, while one was hospital-based.

### Intervention and control group characteristics

Of the nine studies, two studies specifically included a-MCI participants (Sungkarat et al., 2017; Sungkarat et al., 2018), two studies enrolled participants with either MCI or a-MCI (Lam et al., 2011; Lam et al., 2014), while the remaining five studies focused exclusively on MCI cases. Notably, the participants in one study had both MCI and type 2 diabetes.

All nine RCTs implemented TCC as their primary intervention. The specific TCC forms varied across studies: five studies employed the 24-form, two utilized the 10-form, and the remaining two studies implemented the 8-form and standing form respectively. While these forms differ in their specific movements, they all fundamentally represent variations within the TCC system, with only minor technical differences between them.

In terms of intervention design, the experimental groups exclusively received TCC training without any additional multi-component interventions. The control groups received either alternative interventions designed to maintain their regular daily physical activity levels or no intervention at all.

The studies were categorized into short-term and long-term groups based on the significant variation in the duration of TCC interventions. Each session ranged from 30 to 60 min, while the overall training program spanned from 12 weeks to 1 year, with sessions conducted two to four times per week.

### Data extraction and synthesis

The pooled effect sizes were estimated with SMD and 95% CI. p < 0.05 was regarded as a significant improvement for the intervention group compared to the control groups. The meta-analysis was implemented using STATA17.0.

Statistical heterogeneity was evaluated through the application of I^2^ tests. When the I^2^ >50% and p < 0.1, the random-effects model would be employed for analysis. In the nine included RCTs, the experimental groups were administered solely the TCC intervention. It was determined that these trials demonstrated a high degree of clinical similarity (Du et al., 2015).

The data extracted from eligible studies included: (a) basic information (authors, publication year, country); (b) participant characteristics (sample size, population type); (c) intervention parameters (frequency and duration); and (d) cognitive outcome measures, as detailed in Table 1.

**TABLE 1:** Summary of TCC studies including participants with cognitive impairment.

Study and location	Study design	Study population	Intervention groups	Frequency and duration of intervention	Cognitive outcomes measured	Results related to cognitive measures
Li et al., (2023) United States	RCT (n = 213)	community-dwelling adults with MCI age ≥65 y	TCC(n = 107) stretching (n = 106)	all groups: 2 (60-min) sessions per week for 24 weeks	Global Cognition: MoCA; CDR–Sum of Boxes score Executive Function: TMT-B Language: Verbal fluency (Category Fluency for Animals) Memory: DSB Attention: DSF	at 24 weeks TCC showed the improvements in MoCA, CDR-SB, TMT-B, and DSB
Liao et al. (2021) Taiwan China	RCT (n = 20)	adults with MCI in Shin-Kong Wu Ho-Su Memorial Hospital age ≥65 y	a-tDCS + TCC (n = 10) sham + TCC (n = 10)	all groups: 3 (40-min) sessions per week for 12 weeks	Global Cognition: MoCA Executive Function: TMT-AB; VWM; ToL Memory: CVVLT Attention: SCWT	at 12 weeks Mean MoCA: Sham + TCC: 25.3 ± 3.19
Lam et al. (2011) Hong Kong, China	RCT (n = 389)	community-dwelling adults with CDR 0.5 or a-MCI age ≥65 y	TCC (n = 171) Control (n = 218)	TCC: 3 (30 min) sessions per week for 5 months Control: a set of muscle stretching exercise	Global Cognition: CDR; MMSE Language: CVFT Memory: DR, SCC Attention: VS (FB)	at 5th months Significant improvements in CMMSE, ADAS-Cog,DR, Chinese trial A test, CVFT and SCC were found in both groups (paired t-test, p < 0.01) Improvements in VS and CDR sum of boxes scores were observed in TCC only (paired t-tests, p < 0.05)
Lam et al. (2014) Hong Kong China	RCT (n = 389)	community-dwelling adults with MCI or a-MCI age ≥65 y	TCC(n = 171) Control (n = 218)	TCC: 3 (30 min) sessions per week for 12 months Control: a set of muscle stretching exercise	Global Cognition: CDR; MMSE Language: CVFT Memory: DS; DR	at 12 months No significant change in MMSE scores in both groups There were improvements in DS, DR, CVFT, CDR, but differences between groups were not significant
Liu et al. (2022) Taipei China	RCT (n = 34)	community-dwelling adults with MCI age ≥65 y	TCC (n = 17) Control (n = 17)	TCC: 3 (50-min) sessions per week for12 weeks Control: daily physical activities	Global Cognition: MoCA Executive Function: TMT Language: SCWT Memory: CVVLT; Spatial N-back Task Attention: SCWT	at 12 weeks TMTA: TCC vs control, p = 0.006 TMTB: TCC vs control, p = 0.004 Delta TMT: TCC vs control, p = 0.007 SCWT seconds: TCC vs control, p = 0.003
Chen et al. (2023) Fuzhou, Harbin, Shenzhen, and Beijing China	RCT (n = 218)	community-dwelling adults with MCI and T2D age ≥60 y	TCC(n = 107) Control (n = 111)	TCC: 3 (60-min) sessions per week for 24 weeks Control: maintained previous lifestyle All groups were provided with a 30-min diabetes self-management education session, once every 4 weeks for 24 weeks	Global Cognition: MoCA Executive Function: TMT-B Language: BNT, DSST Memory: MQ	at 36 ks Between TCC and Control group: TCC improved mean MQ scores 99.39 [12.70] vs. 92.98 [14.43], respectively; between-group mean difference, 6.41 [95% CI, 2.71–10.11]), DSST scores (33.82 [10.48] vs. 30.70 [9.60], respectively; between-group mean difference, 3.12 [95% CI, 0.46–5.78]), TMT-B scores (187.76 [74.46] vs. 215.53 [74.47], respectively; between-group mean difference, −27.78 [95% CI, −47.91 to −7.64])
Sungkarat et al. (2017) Chiang Mai, Thailand	RCT (n = 66)	community-dwelling adults with a-MCI age ≥60 y	TCC (n = 33) Control (n = 33)	TCC: 3 (50 min) sessions per week for 3 weeks at center-based; 3 (50 min) sessions per week for12 weeks at home-based. Control: received educational material	Executive Function: TMT B-A, DS Memory: DR Attention: BD	at 15weeks TCC performed better in DR, BD, TMT B–A (P < 0.05); Between groups, DS did not differ (P > 0.05)
Sungkarat et al. (2018) Chiang Mai, Thailand	RCT (n = 66)	community-dwelling adults with a-MCI age ≥60 y	TCC (n = 33) Control (n = 33)	TCC: 3 (50 min) sessions per week for 6 months Control: received cognitive education	Executive Function: TMT B-A, DS Memory: DR Attention: BD	at 6 months TCC performed significantly better in the DR and TMT B-A (P < 0.02); Between groups, DS forward/backward scores did not differ (P > 0 0.05)
Hwanget al (2023) Taipei, China	RCT (n = 126)	community-dwelling adults with MCI age ≥65 y	TCC (n = 63) Health Rducation (HE) (n = 63)	TCC: at least 4 (50 min) sessions per week for 24 weeks HE: 1 (45 min) session per week for 24weeks	Global Cognition: MDRS Memory: Memory	at 12 months TCC increase in the MDRS’s

Primary outcomes focused on cognitive function, including domains of global cognition, memory, language, attention, and executive function. Outcome data comprised post-intervention means and standard deviations from both experimental and control groups. Subsequently, the effects of TCC on executive function, memory, and global cognition were synthesized and analyzed in older adults with MCI.

### Quality and risk-of-bias assessments

**FIGURE 2:**
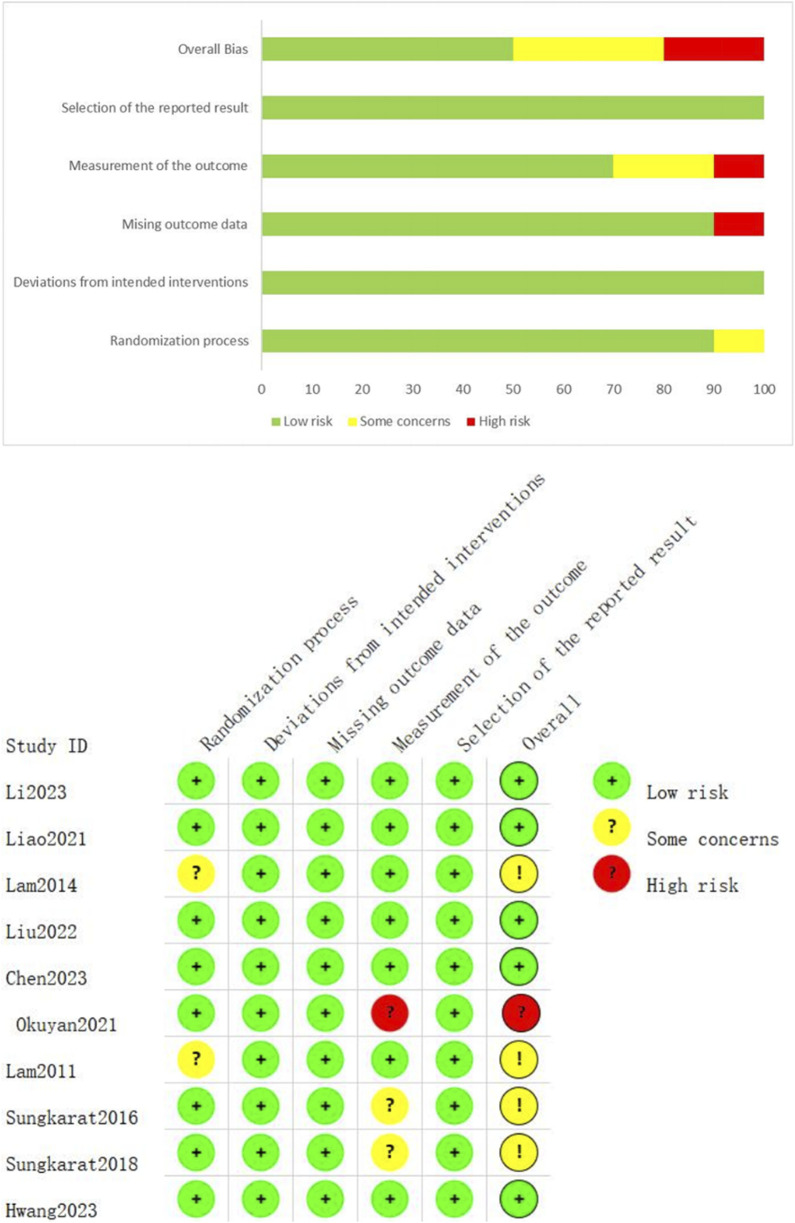
Assessment of methodological quality and risk of bias for RCTs.

The bias in individual studies was assessed using the Cochrane Risk of Bias (2.0) tool. The quality and risk-of-bias assessments required separate evaluations by two reviewers (WH,WWT). Methodological quality and associated risk of bias for all RCTs are summarized in Figure 2.

The funnel plot and Egger’s test were employed to assess publication bias among eligible studies. A random-effects model was employed in the meta-analysis of nine eligible RCTs evaluating the therapeutic efficacy of TCC on global cognition, memory, and executive function in MCI populations. To mitigate publication bias, trials exhibiting significant funnel plot asymmetry (Egger’s test: p < 0.05) were excluded through trim-and-fill analysis.

## Results

### Outcomes measured

The cognitive function outcomes included global cognition, attention, executive function, memory, language, and other domains. The results related to cognitive function were categorized into the following domains: (Park et al., 2023):

1. Global Cognition: Mini Mental Status Exam (MMSE), Alzheimer’s Disease Assessment Scale-Cognitive Subscale (ADAS-cog), Clinical Dementia Rating (CDR), Montreal Cognitive Assessment (MoCA), Mattis Dementia Rating Scale (MDRS);
2. Executive Function: Trail Making Test-B (TMT-B), Trail Making Test B minus A (TMT B-A), Tower of London (ToL);
3. Linguistic Competence: Verbal fluency (Category Fluency for Animals), Category Verbal Fluency Tests (CVFT), Stroop Color and Word Test (SCWT), Boston Naming Test (BNT);
4. Memory: Delayed Recall, Wechsler Memory Quotient (MQ), the Chinese Version of the Verbal Learning Test (CVVLT).

### Quality of studies and risk of bias

A quality assessment of the nine studies using the Cochrane Risk of Bias 2 (RoB2.0) tool revealed that five (55.6%) were low-risk, four (44.4%) raised some concerns, and none were high-risk. Across bias domains:

Randomization process: Seven studies (77.8%) low-risk, and two studies (22.2%) raised some concerns.

Deviation from intended interventions: All nine studies (100%) low-risk.

Missing outcome data: All nine studies (100%) low-risk.

Outcome measurement: Seven studies (77.8%) low-risk, and two studies (22.2%) raised some concerns.

Selection of reported results: All studies low-risk.

### Synthesis of results

Among the nine included studies, seven specifically investigated TCC effects on global cognition in older adults with MCI, while all nine studies systematically evaluated outcomes in executive function and memory. For methodological consistency, pre-post intervention changes were quantified by extracting mean differences (MD) and corresponding standard deviations (SD) between baseline and post-intervention measurements across all cognitive assessments.

A random-effects model was implemented for meta-analysis of the five eligible RCTs assessing TCC efficacy on global cognition in MCI populations. Two trials demonstrating significant funnel plot asymmetry were excluded through trim-and-fill analysis to mitigate publication bias.

**FIGURE 3:**
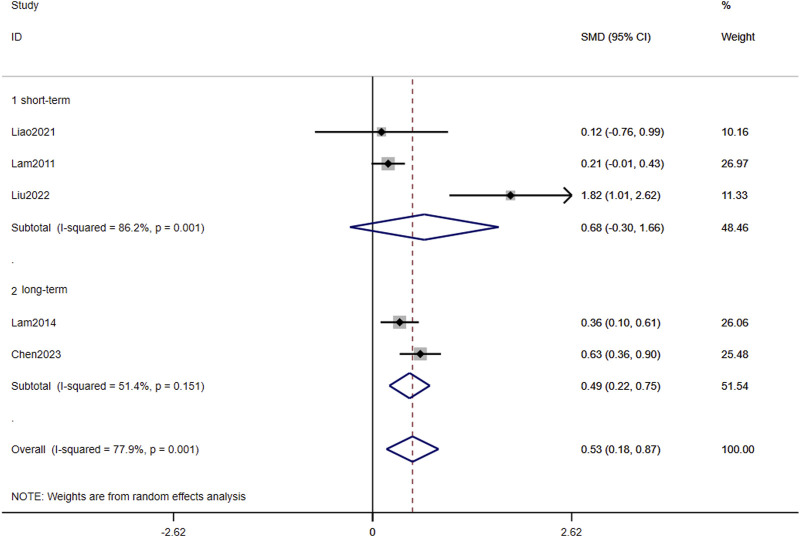
Forest plots of the effects of TCC on global cognition.

**FIGURE 4:**
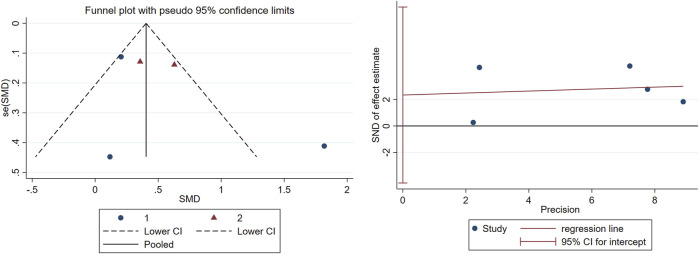
Publication bias in the included studies.

In Figure 3 **,** long-term TCC (p < 0.001, SMD = 0.488, 95%CI: 0.222–0.754) significantly improved global cognition in older adults with MCI, while short-term did not (p = 0.172, SMD = 0.682, 95%CI: −0.397–1.660). Overall, the pooled p-value was 0.003, indicating that TCC significantly improved global cognition. Egger’s linear regression analysis demonstrated no small-study effects (p = 0.345) for global cognition outcomes. The corresponding funnel plot visualization exhibited moderate symmetry in SMD effect size distribution across studies (Figure 4 **)**. This pattern aligns with the Cochrane Collaboration’s criteria for low risk of publication bias.

Following trim-and-fill procedure under the random-effects framework, one outlier study exhibiting significant funnel plot asymmetry was excluded. This sensitivity analysis aligns with PRISMA guidelines for addressing potential publication bias in systematic reviews.

**FIGURE 5:**
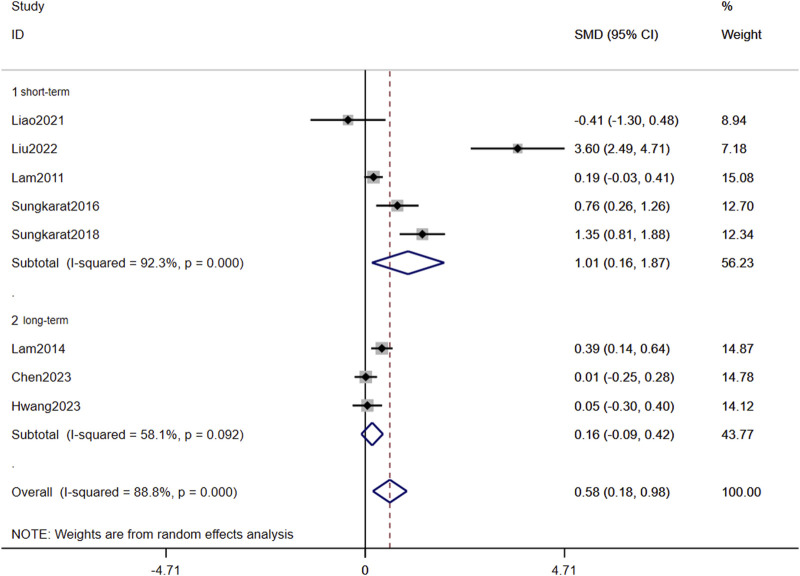
Forest plots of the effects of TCC on Memory.

The meta-analysis incorporating eight studies demonstrated a significant overall effect of TCC on memory improvement in older adults with MCI (pooled p = 0.005). Notably, subgroup analyses revealed differential effects based on intervention duration: short-term TCC training showed statistically significant memory enhancement (p = 0.021, SMD = 1.010, 95%CI: 0.155–1.865), whereas long-term training did not reach statistical significance (p = 0.214, SMD = 0.162, 95% CI: −0.094–0.417) (Figure 5).

**FIGURE 6:**
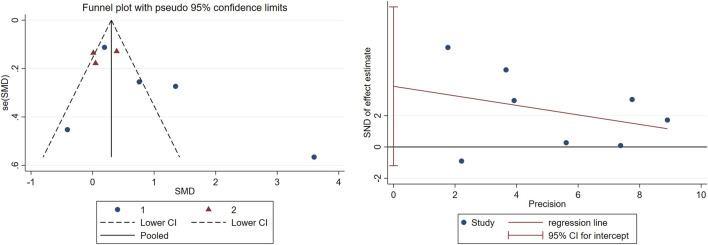
Publication bias in the included studies.

Publication bias was systematically evaluated through funnel plot visualization and Egger’s regression test. The funnel plot exhibited minimal asymmetry, with only one outlier study deviating from the pooled estimate. Egger’s test confirmed no significant publication bias (p = 0.111) (Figure 6).

Following the implementation of the trim-and-fill procedure within a random-effects model, three trials demonstrating substantial funnel plot asymmetry were excluded. Post-adjustment analyses confirmed that TCC interventions sustained statistically significant enhancements in executive function outcomes (pooled p = 0.006).

**FIGURE 7:**
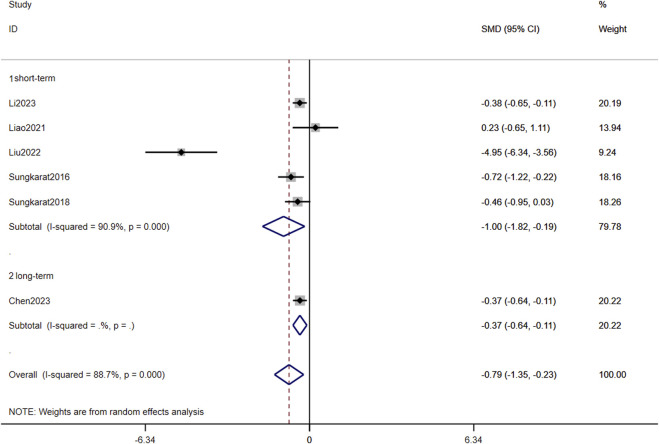
Forest plots of the effects of TCC on executive function.

Sensitivity analyses were conducted, focusing exclusively on six methodologically rigorous studies meeting predefined high-quality criteria. This stratified examination demonstrated consistent therapeutic effects across temporal dimensions: for short-term interventions (≤24 weeks), significant benefits emerged (p = 0.016), with a substantial SMD (SMD = −1.003, 95% CI: −1.817 to −0.188), reflecting large-magnitude improvements. Long-term interventions (>24 weeks) similarly showed durable effects (p = 0.006, SMD = −0.373, 95% CI: −0.641 to −0.105), suggesting dose-response sustainability (Figure 7).

**FIGURE 8:**
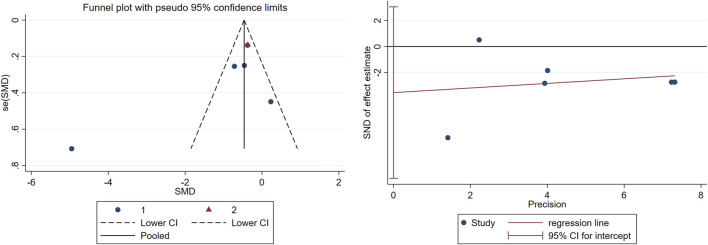
Publication bias in the included studies.

To address potential bias, a *post hoc* Egger’s regression test was conducted after excluding three outlier studies, which indicated no significant publication bias (p = 0.210) (Figure 8).

### Reports of safety and adverse events

Among the nine analyzed studies, eight (88.89%) implemented safety monitoring protocols, with one study (11.11%) omitting this critical component. Six studies documented a complete absence of adverse events in their experimental groups. Of the remaining two studies reporting safety outcomes, one observed adverse events unrelated to the experimental intervention, while the other identified treatment-related adverse reactions in 7 participants (6.5% of the experimental cohort).

## Discussion

Previous studies have consistently highlighted insufficient evidence supporting the efficacy of single-component physical activity interventions for mitigating cognitive decline. Although the results predominantly showed no significant cognitive benefits from interventions consisting solely of physical activity, several cognitive outcomes demonstrated advantages of physical activity over control groups in studies of aerobic training, resistance training, and multi-component physical activity interventions (Brasure et al., 2018).

To our knowledge, this investigation represents the first meta-analysis employing rigorous selection criteria: 1) exclusive inclusion of RCTs implementing TCC as a standalone intervention; 2) strict age stratification (participants ≥60 years with MCI); and 3) standardized statistical methodology using Stata 17.0. Our three-level meta-analytic approach evaluated TCC’s effects across global cognition, memory, and executive function. Regarding executive function, although previous narrative and meta-analytic reviews have demonstrated the effect of exercise training on executive function broadly; the strength of this evidence was under debate (Diamond and Ling, 2016). The current findings substantiate these observations by demonstrating that both short-term (≤6 months) and extended (>6 months) TCC practice significantly enhances executive performance in older adults with MCI.

### TCC for global cognition

This meta-analysis incorporated five RCTs assessing global cognitive function using standardized instruments (MMSE or MoCA). Duration-stratified subgroup analyses revealed differential efficacy patterns: interventions were classified as short-term (10–24 weeks; n = 3 studies) or long-term (25–52 weeks; n = 2 studies). Pooled estimates demonstrated that prolonged TCC practice significantly enhanced global cognition compared to controls, with robust effect sustainability confirmed through funnel plot symmetry and non-significance of Egger’s regression intercept (Figures 3, 4). Conversely, short-term intervention failed to produce significant improvements. The dose-response relationship between intervention duration and cognitive outcomes reached statistical significance.

These findings provide robust evidence that sustained TCC practice induces clinically meaningful improvements in global cognition among older adults with MCI. A previous systematic review based on seven RCTs proposed that changes in cognitive function during TCC training may be associated with structural and functional changes in the cortex related to cognition (Lin et al., 2021). One of the common mechanisms underlying exercise-induced cognitive benefits is the facilitation of neuroplasticity in specific brain structures through physical activity (Hötting and Röder, 2013). In individuals aged 60 years and older with MCI, sustained TCC practice functions as a neurostimulatory intervention that induces cerebral structural remodeling. This neurobiological mechanism is mediated through enhanced functional connectivity between the prefrontal cortex (PFC), motor cortex (MC), and occipital cortex (OC), which subsequently modulates myogenic regulation, sympathetic nervous system activity, and endothelial cell metabolic processes (Xie et al., 2019). The absence of short-term therapeutic effects may be predominantly attributable to neurobiological mechanisms that require prolonged intervention for measurable outcomes to emerge. These findings may provide evidence-based guidance to clinicians on using TCC to enhance global cognition in individuals with MCI aged 60 years or older.

### TCC for memory

TCC requires substantial cognitive effort to remember and perform movements, which may prevent or alleviate memory loss in older adults with MCI. TCC involves learning and memorizing new skills and movement patterns. Studies of other skill-based learning activities, such as dance, juggling, and music, have shown improvements in cognitive function and underlying neural mechanisms (Kattenstroth et al., 2010). Although the cited studies utilized simplified TCC protocols, the intervention maintained essential elements, such as continuous motion patterns and divided-attention requirements, thereby sustaining cognitive demands on participants’ working memory and procedural recall. To validate this interpretation, we conducted a subgroup analysis examining TCC’s effects on memory, which demonstrated significant improvements following short-term interventions, whereas no statistically significant progress was observed in long-term implementations (Figures 5, 6).

Research findings on the impact of physical activity exhibited variability across global cognitive assessments and domain-specific cognitive measures (Chen et al., 2023; Nguyen and Kruse, 2012). One study reported no significant associations between TCC and memory enhancement in older adults with cognitive impairment compared to a health education control group (Wang et al., 2018). Furthermore, correlation analyses specific to the TCC cohort revealed a negative association between executive attention capacity and total accumulated TCC practice hours (Wei et al., 2014).

TCC intervention induces structural and functional reorganization in key neural substrates, particularly enhancing cortical neuroplasticity markers (e.g., thickness, connectivity, homogeneity) and optimizing executive control networks (Pan et al., 2018). TCC induces neuroplastic reorganization in brain function through specific cortical activation patterns, and intervention duration exhibits potential correlations with the degree of neural mechanism optimization. This confirms the duration-dependent neurocognitive effects of TCC, particularly in enhancing memory function.

A-MCI, a clinical subtype of MCI, is characterized by multidomain memory decline (Wang et al., 2023). The four included trials enrolled participants diagnosed with a-MCI, with outcome measures encompassing memory performance and executive function. The findings demonstrated significant duration-dependent correlations in the therapeutic benefits of TCC (Oh et al., 2023).

### TCC for executive function

Current evidence indicates exercise training interventions specifically enhance executive function, as documented in prior meta-analyses (Northey et al., 2018). However, the differential mechanisms through which exercise may improve cognitive performance in adults aged ≥60 years with MCI remain underexplored. Although narrative reviews and meta-analytic studies have demonstrated exercise-induced improvements in executive function, concerns persist regarding the methodological robustness of the supporting evidence. Executive function was systematically assessed using the TMT-B, the predominant neuropsychological instrument, across six qualified studies. Subgroup analyses stratified by intervention duration revealed significant executive function improvements following both short-term and long-term TCC interventions (p = 0.006) (Figures 7, 8). This meta-analysis conclusively demonstrates that TCC interventions produce clinically meaningful executive function enhancements in older adults with MCI (≥60 years).

Exercise training demonstrates consistent enhancements in executive function across all demographic parameters (e.g., frequency, intensity, modality, session duration, program length, and sex), except in populations exceeding 75 years of age (Chen et al., 2020). A pivotal finding from Li et al. (2023) — one of the included trials—revealed superior executive function performance in the intervention cohort compared to controls. The intervention group’s mean age was 75.9 years.

The study implemented the TMT-B for executive function assessment, employing a standardized protocol involving sequential practice of eight TCC postures guided by audiovisual cues. The 24-week intervention protocol comprised biweekly, 60-min training sessions. Systematic adverse event monitoring during the trial period identified six documented incidents, none of which were related to the intervention. Crucially, these findings provide empirical evidence supporting TCC’s capacity to improve executive function in MCI patients aged over 75 years, specifically in participants with a mean age exceeding typical age-related efficacy thresholds.

### Limitations and suggestions for future research

This study has two main limitations due to the limited number of RCTs that met the inclusion criteria.

First, the therapeutic mechanisms underlying TCC interventions targeting a-MCI, a core sub-type of MCI, still require validation through longitudinal studies with larger sample sizes.

Second, as a critical covariate, intervention duration exerts direct effects on cognitive assessment outcomes, particularly on memory function. This necessitates more granular investigations to delineate temporal thresholds for memory enhancement and establish standardized temporal effect models, thereby informing the optimization of therapeutic protocol design in future research.

## Conclusion

Current meta-analytic evidence establishes a robust duration-dependent therapeutic relationship between TCC practice and cognitive enhancement in older adults (≥60 years) with MCI, particularly in the domains of global cognition and memory. Sustained TCC practice (>6 months) induces clinically meaningful improvements in global cognition, while memory outcomes exhibit nonlinear temporal dynamics characterized by an initial phase of rapid improvement (short-term acceleration) followed by a stabilization phase (long-term plateau). Subgroup analyses stratified by intervention duration further revealed significant and comparable improvements in executive function across both short-term (≤6 months) and long-term (>6 months) TCC interventions, indicating temporal invariance in executive function benefits.

## Data Availability

The original contributions presented in the study are included in the article/supplementary material, further inquiries can be directed to the corresponding author.

## Abbreviations

MCI, Mild Cognitive Impairment; TCC, Tai Chi Chuan; RCTs, Randomized Controlled Trials; SMD, Standardized Mean Differences; AD, Alzheimer’s Disease; CKNI, China National Knowledge Infrastructure; a-MCI, mnestic multiple-domain Mild Cognitive Impairment; BD, Block Design; BNT, Boston Naming Test; CVFT, Category Verbal Fluency Tests; DS, Digit Span; DR, Delay Recall; DSST, Digit Symbol Substitution Test; DSB, Digit Span Backward; DSF, Digit Span Forward; MDRS, Mattis Dementia Rating Scale; MMSE, Mini-Mental State Examination; SCWT, Stroop Color and Word Test; SCC, Subjective Cognitive Complaints; ToL, Tower of London; CVVLT, The Chinese Version of the Verbal Learning Test; TMT-B, Trail Making Test-B; TMT B-A, Trail Making Test B minus A; VS(FB), Visual span (forward), Visual span (backward); VWM, Visual Working Memoryy; MQ, Wechsler Memory Quotient.
